# Coastal urbanization alters carbon cycling in Tokyo Bay

**DOI:** 10.1038/s41598-020-77385-4

**Published:** 2020-11-23

**Authors:** Atsushi Kubo, Jota Kanda

**Affiliations:** 1 Department of Geosciences, Shizuoka University, 856 Ohya, Suruga-ku, Shizuoka city, Shizuoka 422-8529 Japan; 2 Department of Ocean Sciences, Tokyo University of Marine Science and Technology, 4-5-7 Konan, Minato-ku, Tokyo 108-8477 Japan

**Keywords:** Carbon cycle, Biogeochemistry

## Abstract

The carbon budget of Tokyo Bay, a highly urbanized coastal basin, was estimated using a box model that incorporated inorganic and organic carbon data over an annual cycle (2011–2012). The surface water represented net autotrophic system in which the annual net community production (NCP) was 19 × 10^10^ gC year^−1^. The annual loading of dissolved inorganic carbon and total organic carbon (TOC) from freshwater inputs was 11.2 × 10^10^ and 4.9 × 10^10^ gC year^−1^, respectively. The annual TOC sedimentation rate was 3.1 × 10^10^ gC year^−1^, similar to the annual air–sea CO_2_ uptake (5.0 × 10^10^ gC year^−1^). Although the NCP and TOC loading from freshwater inputs were respectively 3.0 and 2.7 times lower than those in the 1970s, the TOC sedimentation rate was similar. Therefore, a relatively high carbon efflux from Tokyo Bay likely occurred in the 1970s, including CO_2_ efflux to the atmosphere and/or export of labile organic carbon to the open ocean. The changes in carbon flow between the 1970s and 2011–2012 resulted from improved water quality due to increased sewage treatment facilities and improved sewage treatment efficiency in the catchment, which decreased the amount of labile organic carbon flowing into the bay.

## Introduction

Coastal waters are dynamic systems because of the interactions between the atmosphere, land, rivers, and open ocean. These waters comprise only a small area of the ocean and have dynamic biogeochemical cycles^1^. Coastal waters receive huge inputs of terrestrial organic and inorganic matter through river discharge and thus constitute one of the most biologically productive areas of the marine hydrological system, exchanging large amounts of organic and inorganic matter with the open ocean^1^. In addition, coastal waters contribute to the global air–sea CO_2_ exchange because of its high biological activity^2,3^. Most coastal waters are significant sources of atmospheric CO_2_ because of terrestrial inorganic carbon input and organic carbon mineralization. In contrast, highly eutrophicated environments are sinks for atmospheric CO_2_, because CO_2_ uptake during photosynthesis exceeds CO_2_ produced during mineralization^4,5^.

Eutrophication contributes to the carbon budget, especially in terms of coastal ocean acidification. Excessive nutrient loads can induce massive algal blooms and subsequent deposition of organic carbon in bottom waters, where organic matter decomposition consumes dissolved oxygen (DO) and produces CO_2_^6^. The formation of both anoxic water masses and ocean acidification greatly impacts marine ecosystems^7–9^. Previous biogeochemical research on coastal waters has mainly investigated nutrient and DO concentrations^10–12^. Despite its potential importance, carbon cycling in coastal waters has only recently been investigated^13^. Moreover, most studies focused on pristine coastal waters that have not been heavily impacted by human activity^13^.

Recently, fluvial dissolved organic carbon (DOC) concentrations in the River Thames, UK, increased significantly because of increased sewage treatment plant (STP) effluent^14^. STP effluents in highly urbanized coastal waters is a source of dissolved inorganic carbon (DIC), nutrients, and organic matter^15,16^. Because information on the flow of inorganic and organic carbon in urbanized coastal waters is increasingly being reported, the impacts of human activities on coastal waters are becoming clearer. However, few studies have estimated carbon cycling and budget in highly urbanized coastal waters^17^. Carbon cycling in coastal ecosystems is increasingly subject to human influences, such as land and water use changes and increased waste and pollution^18^.

Most megacities, defined as metropolitan areas with a total population of greater than 10 million people, are located in coastal areas, and the number of megacities globally is likely to increase from 23 to 37 by 2025^19^. Moreover, approximately 40% of the world’s population is settled in coastal zones, and developed urban areas with complete sewer systems are expected to expand rapidly^19^. Research on the coastal carbon budget in areas around megacities could improve understanding of carbon cycling in coastal waters and project potential future influences of coastal urbanization.

Tokyo Bay is surrounded by Tokyo metropolitan area, the world’s largest megacity. The nutrient concentration in the bay increased markedly with urbanization of the watershed between the 1950s and 1980s but gradually decreased thereafter because of advances in effluent treatment and sewage treatment efficiency^11^. Carbon cycling in Tokyo Bay may have changed with this decrease in nutrients. In this study, we observed annual DIC, DOC, and particulate organic carbon (POC) in Tokyo Bay to evaluate the carbon budget based on a box model. Nutrient budget estimates were also obtained from this model, based on monthly observation data from Tokyo Bay^20^. We compared our results with those of carbon fluxes obtained from research in the 1970s^20–22^. The aim of this study was to elucidate changes in carbon cycling in the highly urbanized coastal waters of Tokyo Bay.

## Study area and methods

### Site description

Tokyo Bay, with a mean water depth of 19 m and area of approximately 920 km^2^, in central Japan is surrounded by the Tokyo metropolitan area, the largest megacity in the world, with a total population of approximately 31 million. The bay has been severely eutrophicated since the late 1950s because of high organic matter input via human activities during the 1950s and 1960s^23^. However, in 1970, a law restricting organic pollutant discharge was enacted. Technical advances, such as the use of phosphorus-free detergents and improved sewage treatment, also reduced the discharge of organic pollutants and nutrients into the bay^24^.

As a result, chemical oxygen demand loading in the bay has decreased significantly from 477 to 183 t per day between 1980 and 2010^25^, and organic carbon concentrations have likewise decreased from the late 1970s to the 2010s^23,26^. Furthermore, ammonium, nitrate, nitrite, and phosphate concentrations in Tokyo Bay have decreased between 1989 and 2015 because of advances in effluent treatment and sewage treatment efficiency^9^. However, nutrient concentration is not a limiting factor in phytoplankton growth, and blooms have continued to occur mainly during spring and summer. Consequently, the bay is a net CO_2_ sink because CO_2_ consumption during photosynthesis exceeds CO_2_ supply from terrestrial organic carbon degradation in the system^27^.

## Methods

**Figure 1:**
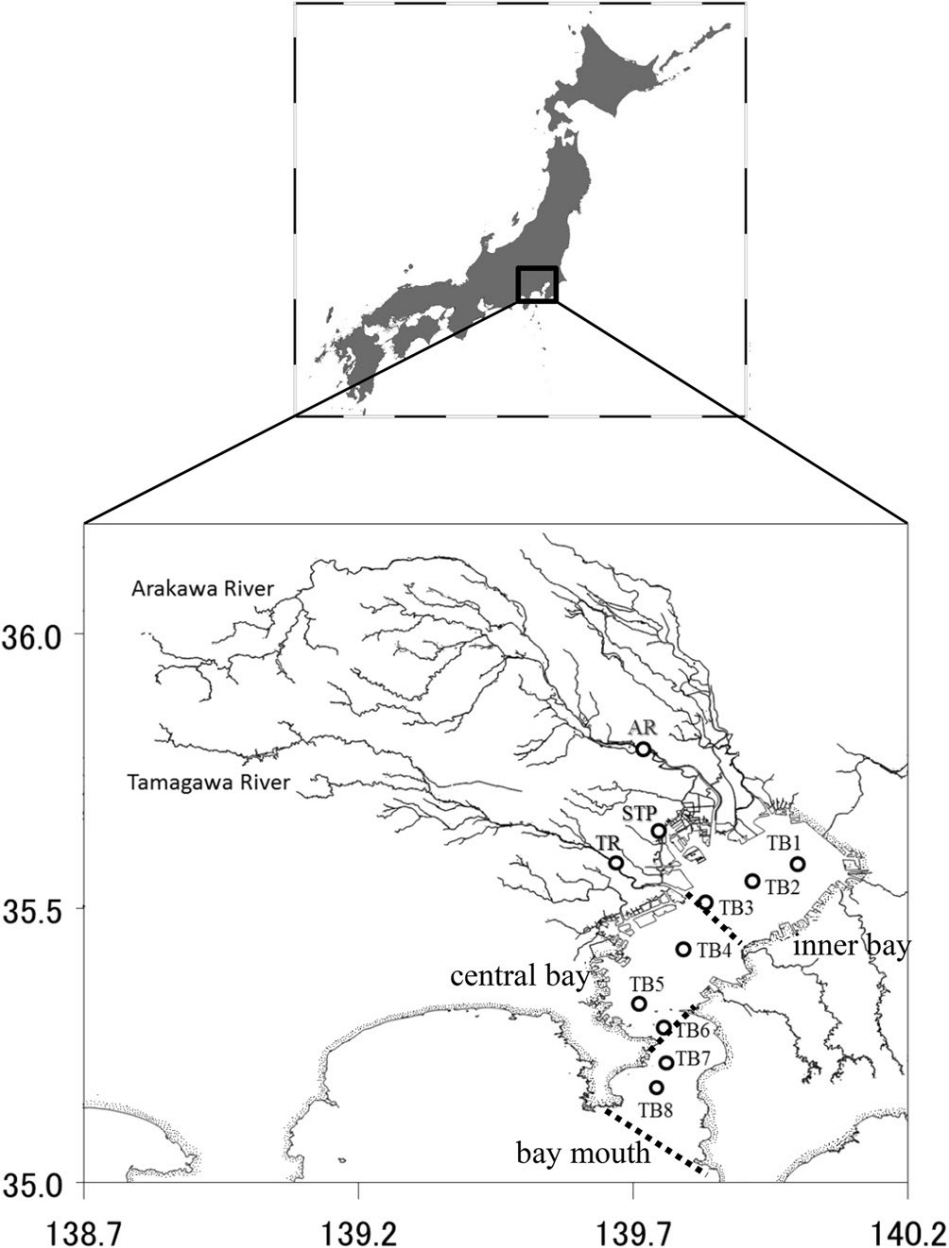
Study area in Tokyo Bay. Black circles indicate sampling locations. Broken lines show box-model cross-sections. The map was created using Surfer (version 13.6.618; https://www.goldensoftware.com/products/surfer).

Monthly sampling from May 2011 to May 2012 was conducted in three freshwater sites and eight stations in Tokyo Bay. The freshwater sampling sites were located at the effluent outflow of the Shibaura STP, lower Tamagawa River, and lower Arakawa River (Fig. 1). Surface samples were collected using a bucket after twice co-wash. Salinity and temperature were measured in the field using an electrical conductivity and temperature meter (EC 300, YSI/Nanotech Inc.).

Between May 2011 and May 2012, seawater samples were collected monthly from the eight stations in Tokyo Bay (Fig. 1) on the R/V *Seiyo-maru*. The CTD (Falmouth Scientific Inc.) was lowered to within 3 m of the seabed. Water samples were taken at 5 m intervals from the surface to a depth of 30 m and at 10 m intervals at depths below 30 m. The DO concentration was measured using an oxygen sensor (RINKO-III, JFE Advantech) onboard the CTD. Water samples for chlorophyll *a* (Chl *a*) analysis were filtered through GF/F filters. After filtration, phytoplankton pigments were extracted using *N*,*N*-dimethylformamide, and the Chl *a* concentrations were determined using the fluorometric method^28^ (TD-700, Turner Designs).

The water samples for DIC analysis were collected without being filtered in 40 mL glass vials, fixed with 150 μL HgCl_2_, and stored at room temperature until analysis. The samples were analyzed with a total organic carbon (TOC) analyzer (TOC-V_CSH_; Shimadzu) within 24 h. The standard deviation of duplicate samples was 2.4 μmol L^−1^ (n = 10). This precision was adequate for transects and profiles of DIC in systems with moderate to large DIC variations, but the accuracy of the coulometric method was less than 1.0 μmol L^−1^^29^. The measured concentrations were calibrated using DIC reference material (The General Environmental Technos).

The TOC concentrations were calculated by summing POC and DOC concentrations. The water samples for DOC analysis were filtered through pre-combusted GF/F filters (450 °C for 3 h) and collected in 40 mL glass vials with silicone/PTFE septa. These samples were acidified with 300 μL 6 mol L^−1^ HCl and stored at 5 °C. DOC analysis was performed using the abovementioned TOC analyzer. Each sample was injected at least three times. The relative standard deviation was < 2%. The filters for the DOC samples were used to measure POC concentrations and stable carbon isotopes of particulate organic matter (δ^13^C_POM_). The sample filtration volume ranged from 50 to 250 mL, and filter samples were frozen at − 80 °C. The POC and δ^13^C_POM_ samples were dried at 60 °C and acidified with HCl vapor to remove carbonates before measurement. POC and δ^13^C_POM_ were measured using a Hydra 20–20 isotope ratio mass spectrometer coupled to an ANCA-GSL elemental analyzer (SerCon Ltd.). Analytical precision for POC and δ^13^C_POM_ was < 0.2% and < 0.08‰, respectively. Novak et al.^30^ reported that DOC was retained on the GF/F filter, and POC was thus overestimated. POC in this study was overestimated by a maximum of 1 μmol L^−1^, because the sample filtration volume was 250 mL at most. However, the TOC estimation error was small, since the same filter was used for DOC and POC sample collection. The POC and DOC concentration data in this study comprised uncorrected values.

Monthly air–sea CO_2_ fluxes, estimated using Wanninkhof’s^31^ equation, was obtained from Kubo et al.^27^. The partial pressure of CO_2_ was measured using a nondispersive infrared sensor analyzer (LI-820, Li-Cor) and membrane equilibrator, which was composed of multilayered composite hollow-fiber membrane modules^32^ (MHF module, Mitsubisi Rayon Co., Ltd.). The response time and standard error of this system were approximately 100 s and < 0.4 μatm, respectively.

The volumes of the monthly river discharge (https://www1.river.go.jp) and STP effluents (Japan Sewage Works Association, 2010) were obtained from statistical data. Monthly DIC and TOC loads from rivers and STP effluents were estimated based on the average concentrations of the lower Arakawa and Tamagawa Rivers and their corresponding discharge rates, along with the Shibaura STP effluents and discharge rate. Rooftop rainwater samples for TOC analysis were collected in May and September 2011 in Shinagawa, Tokyo. Precipitation data were obtained from the Japan Meteorological Agency (https://www.jma.go.jp/jma/index.html). The monthly TOC loads from rainwater were estimated based on the average rainwater TOC concentration and total monthly precipitation.

**Figure 2:**
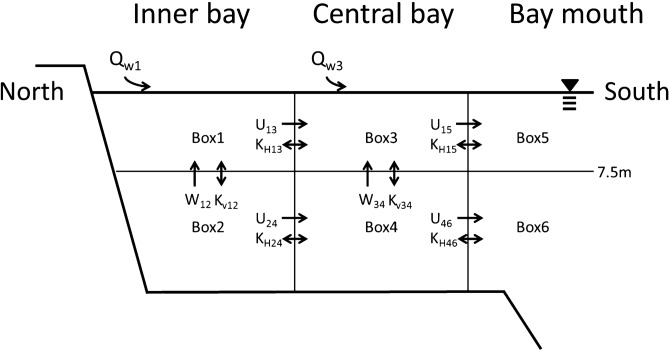
Cross-section of box model in Tokyo Bay. Q indicates amount of supply from rivers and sewage treatment effluent, U and W indicate horizonal and vertical flow velocity, respectively, and K is diffusion coefficient (see Supplementary Information for more details on calculation of each parameter).

A simple advective–diffusive box model^20^ was used to estimate the carbon budget for Tokyo Bay (see Supplementary Information for details). In this model, Tokyo Bay was divided into two layered boxes (surface and bottom layers) based on three vertical cross-sections (inner bay, central bay, and bay mouth; Fig. 2). The surface layers were 7.5 m deep^20^, because the vertical circulation flow rates did not change significantly when the boundary layer varied from 5 to 15 m in a two-layer box model of Tokyo Bay^33^.

## Results and discussion

At the freshwater sampling sites, DIC, DOC, POC, and δ^13^C_OM_ concentrations were significantly lower than those in Tokyo Bay (t-test, P < 0.05; Tables S1–S3, Fig. S1). These parameters did not show clear seasonal patterns at the freshwater sampling sites. DIC and Chl *a* were significantly negatively correlated (t-test, P < 0.05), and a positive correlation existed between POC and Chl *a* (t-test, P < 0.05). At the Shibaura STP, Chl *a* and δ^13^C_POM_ were constant throughout the year. DOC concentrations were significantly higher than those in Tokyo Bay (t-test, P < 0.05), and the highest concentration was observed at the Shibaura STP (Tables S1–S3, Fig. S1). The spatial distributions of DO, temperature, and salinity in the bay are shown in Fig. S1a–c, respectively.

Temperature was higher and salinity was lower in the surface water of the inner bay than in that of the central bay and bay mouth throughout the year. Moreover, clear seasonal stratification occurred between May and September 2011. DO concentrations in the bottom water of the inner bay gradually decreased to nearly zero between June and September 2011, and a widespread hypoxic bottom water mass was observed in August and September 2011, which extended throughout the bay. In contrast, during December and February 2011, the water column in these two regions was well-mixed vertically from the surface to the seabed.

The spatial distributions of Chl *a*, DOC, POC, and δ^13^C_POM_ are presented in Fig. S1d–g, respectively. These values were higher in summer than in winter in the bay surface water. The concentrations of these parameters in the surface water of the inner bay were high and decreased towards the bay mouth. In contrast, these concentrations were almost constant in the bottom water, although DOC was slightly higher in the inner bay than the bay mouth throughout the year. Furthermore, the concentrations of the parameters varied significantly between stations TB6 and TB7 in January to March (Fig. S1). This was because a thermohaline front existed at the bay mouth during winter and early spring^34^. Lower DIC concentrations were observed in summer than in winter, and higher concentrations were observed in the bottom water throughout the year, especially during summer (Figure S1h). DIC concentrations in the surface water were low in the inner bay and increased towards the bay mouth. Typical δ^13^C_POM_ values of marine POC range from -22 to -18‰ and are higher than terrestrial δ^13^C_POM_ (− 33 to − 25‰)^35^. In the bay surface water, the annual average δ^13^C_POM_ was − 18.8‰ and was mostly higher than the terrestrial values (− 25‰) throughout the year. Therefore, POC in the bay water was dominated by marine-derived organic carbon.

The annual DIC and TOC loadings from river water, including STP effluent, into the bay were 11.2 × 10^10^ and 4.9 × 10^10^ gC year^−1^, respectively. The summer and spring DIC and TOC loading amounts (1.1 × 10^10^ and 0.48 × 10^10^ gC month^−1^, respectively) were higher than those during autumn and winter (0.77 × 10^10^ and 0.33 × 10^10^ gC month^−1^, respectively). The average TOC concentration of rainwater was 50 μmol L^−1^, which was similar to that of global rainwater^36^, and the annual TOC loading from rainwater was 8.4 × 10^8^ gC year^−1^. The TOC loading from rain during spring and summer (0.85 × 10^8^ gC month^−1^) was double that during autumn and winter (0.55 × 10^8^ gC year^−1^). However, this effect was negligible, as it was two orders lower than the other carbon flows in Tokyo Bay.

**Figure 3:**
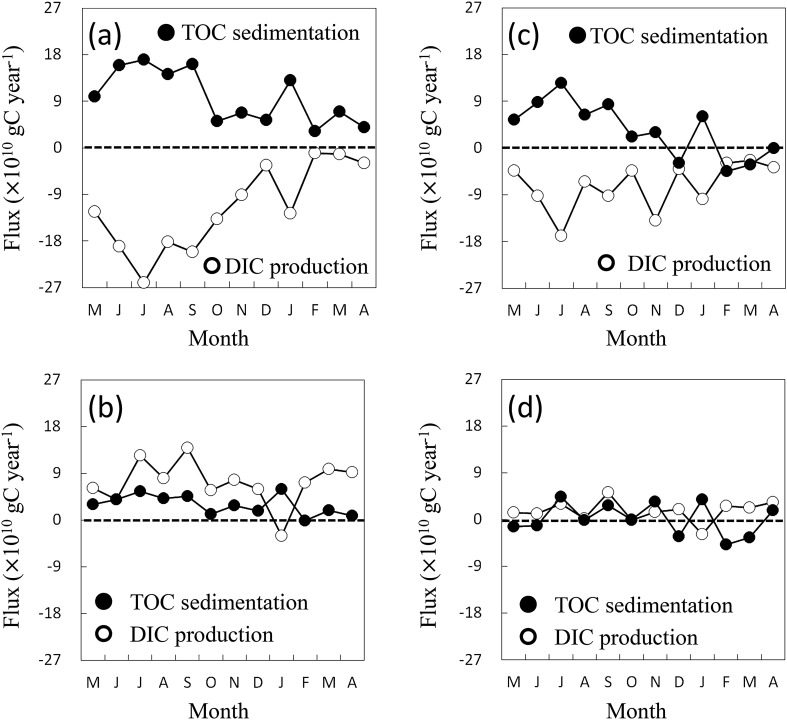
Monthly flux of total organic carbon (TOC) sedimentation rate (black circle) and dissolved inorganic carbon (DIC) production rate (white circle) in each box from May 2011 to April 2012: (**a**) Box 1, (**b**) Box 2, (**c**) Box 3, and (**d**) Box 4. Positive TOC sedimentation rate indicates TOC sedimentation from surface to bottom water or from bottom water to sediment. Negative TOC sedimentation rate indicates resuspension of TOC. Positive DIC production rate indicates DIC production in box, and negative value indicates DIC uptake in box.

Figure 3 presents the monthly variations in the DIC production and TOC sedimentation rates for each box in the model. In the surface waters (Boxes 1 and 3), the DIC production value was negative throughout the year, as DIC was consumed by phytoplankton. Strongly negative DIC values were found in the inner bay (Box 1) from May to October. A negative correlation existed between the DIC production and TOC sedimentation rates in the surface waters (*R*^2^ = 0.79, P < 0.001), suggesting that TOC was produced via active photosynthesis and exported to the bottom water.

Conversely, in the bottom water, the DIC production rate was positive throughout the monthly observation data (Boxes 2 and 4) as a result of TOC decomposition. TOC sedimentation occurred throughout the year in most parts of the bay, except during winter in the central bay area. During spring and summer, the TOC sedimentation rate was high in the inner bay (Box 2), whereas TOC was resuspended in the central bay during winter (Box 4). This seasonal pattern was also observed in the nitrogen and phosphorus budgets in a previous box model analysis of the bay^20^.

**Figure 4:**
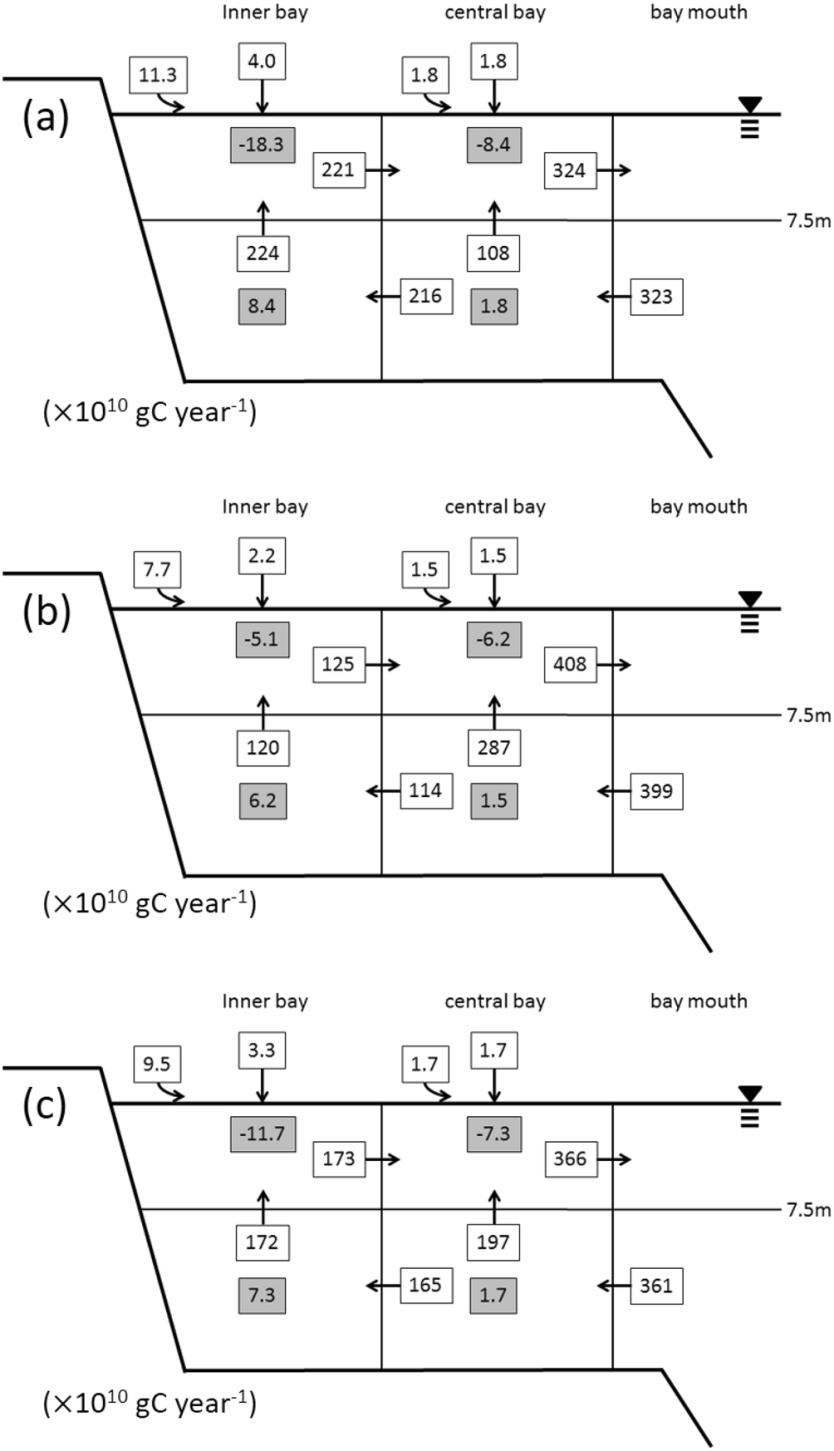
Average dissolved inorganic carbon (DIC) flow in Tokyo Bay during (**a**) May and October (stratified season), (**b**) November and April (vertically mixed season), and (**c**) entire year. Numbers with black and gray squares denote each carbon flux (× 10^10^ gC year^−1^). Curved and straight arrows from outside box (Boxes 1 and 3) indicate DIC input from land and air–sea exchange of CO_2_, respectively. Numbers with gray square indicate DIC production (positive value) or uptake (negative value).

The DIC flows in Tokyo Bay are shown in Fig. 4. The annual average net community production (NCP; DIC production rate at the surface waters; see Supplementary Information for more detail) in the surface water was 19 × 10^10^ gC year^−1^, corresponding to 207 gC m^−2^ year^−1^. NCP was higher during spring and summer (2.23 × 10^10^ gC month^−1^) than during autumn and winter (0.94 × 10^10^ gC month^−1^). In the bottom water, the DIC production rate was 9.0 × 10^10^ gC year^−1^ and was higher during spring and summer (0.85 × 10^10^ gC month^−1^) than during autumn and winter (0.64 × 10^10^ gC month^−1^).

NCP measurements have hitherto not been conducted in Tokyo Bay. Therefore, the ratio of the gross primary production (GPP) to NCP was used to compare the NCP obtained in this study. The GPP/NCP ratio in Tokyo Bay was 1.85 in 1974 and 1.92 in 1994^37^, slightly lower than that found in other studies on autotrophic coastal water (approximately 2.0)^38^. If a GPP/NCP ratio of 2.0 was assumed, the extrapolated GPP in this study was 414 gC m^−2^ year^−1^, which was lower than the GPP values in the 1970s and 1980s but similar to those from the 1990s (Table 1). This supports previous findings that surface-water Chl *a* concentrations decreased between the 1970s and 1980s^39^ but remained constant between the 1990s and 2015^7^: Annual mean Chl *a* concentrations were 40–50 μg L^−1^ in the 1970s and 1980s^39,40^ and decreased to 26.3, 23.7, and 27.4 μg L^−1^ in the 1990s, 2011, and 2012, respectively^11^.

**Table 1:** Primary production in Tokyo Bay (gC m^−2^ year^−1^).

Observation year	Primary production	Method	Reference
1972	1242	^14^C method	Funakoshi et al.^41^
1988	2044	^14^C method	Yamaguchi et al.^42^
1989	1242	^14^C method	Yamaguchi et al.^42^
1997	414	^13^C method	Bouman et al.^43^
1998	522	^13^C method	Bouman et al.^43^
2002	500	^13^C method	Itoh^44^
2011	414	Box model	This study

Nutrients and organic carbon inputs from terrestrial sources have also decreased significantly from the 1970s to 2015^11,23,26^. Annual mean dissolved inorganic nitrogen and phosphate concentrations were about 40 and 1.5 μmol L^−1^, respectively, during the 1970s and 1980s^42^. Although dissolved inorganic nitrogen concentrations increased to 54 μmol L^−1^ in the 1990s, it decreased to 30.5 μmol L^−1^ in 2011. In contrast, phosphate concentrations decreased to 1.1 and 0.7 μmol L^−1^ in the 1990s and 2011, respectively^11^. Decreasing nutrient loads from the rivers reduced primary production in the bay^42,43^. Nevertheless, GPP in Tokyo Bay was still higher than that of most coastal waters globally^45^. The bay is therefore a strong net sink for atmospheric CO_2_, because the consumption of CO_2_ during photosynthesis exceeded the supply from terrestrial organic carbon degradation in the system^27^.

Some studies have reported that urbanized coastal water was a net sink for atmospheric CO_2_, although most coastal water was a significant net source for CO_2_^4,46^. For example, Aby Lagoon in Ivory Coast and Guanabara Bay in Rio de Janeiro, Brazil, are surrounded by populated areas. In these cases, CO_2_ consumption was exacerbated by a massive influx of nutrients and high biological activity^4,47^. The annual mean concentrations of dissolved inorganic nitrogen, phosphate, and Chl *a* in central Guanabara Bay were 12.4 μmol L^−1^, 1.5 μmol L^−1^, and 57.6 μmol L^−1^, respectively^4^, and were comparable to the levels measured in Tokyo Bay.

**Figure 5:**
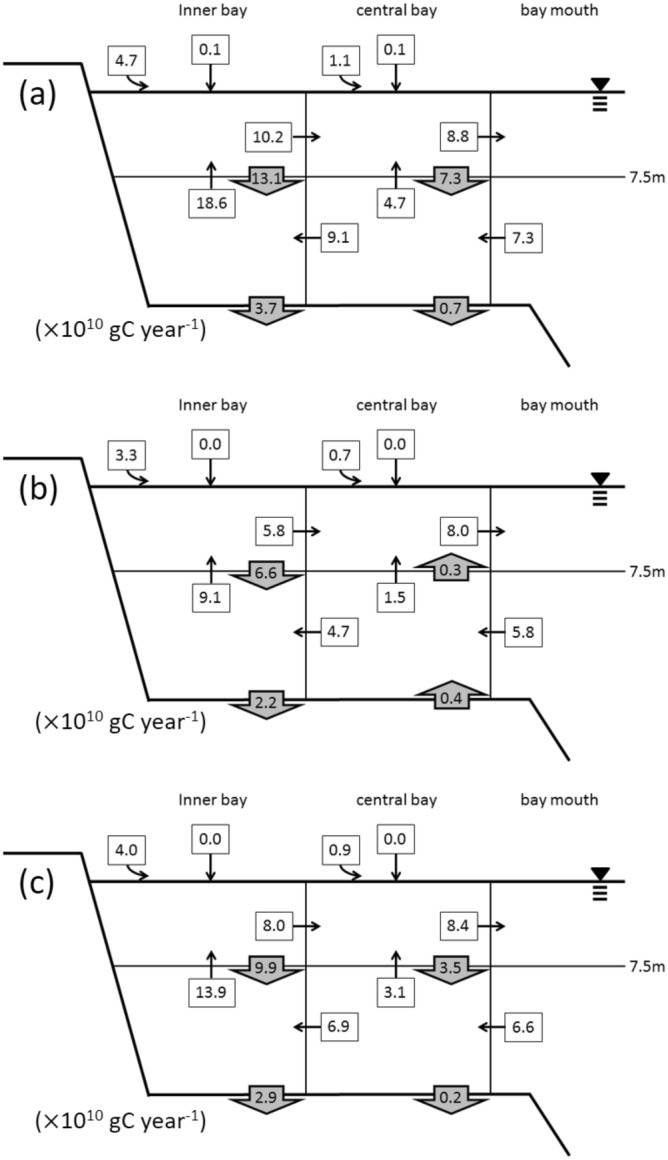
Average total organic carbon (TOC) flow in Tokyo Bay during (**a**) May and October (stratified season), (**b**) November and April (vertically mixed season), and (**c**) entire year. Numbers with black and gray squares denote each carbon flux (× 10^10^ gC year^-1^). Curved and straight arrows from outside box (Boxes 1 and 3) indicate TOC input from land and TOC precipitation, respectively. Numbers with gray square indicate TOC sedimentation (downward arrow) or resuspension (upward arrow).

The TOC flow in Tokyo Bay is shown in Fig. 5. The average annual TOC sedimentation rate from the surface to the bottom water was 13.4 × 10^10^ gC year^−1^, with higher values observed in spring and summer (1.70 × 10^10^ gC month^−1^) than in autumn and winter (0.53 × 10^10^ gC month^−1^). This resulted from nutrient- and TOC-rich discharge from rivers and increased primary production during spring and summer. The TOC sedimentation rate from the bottom water to the sediment was also higher during spring and summer (0.37 × 10^10^ gC month^−1^) than during autumn and winter (0.15 × 10^10^ gC month^−1^). The annual mean TOC sedimentation rate was 3.1 × 10^10^ gC year^−1^, slightly lower than the export rate reported for the bay in 1980^22^ (4.2 × 10^10^ gC year^−1^). TOC sedimentation rates in this study included the annual DOC efflux from sediment pore water, which was 0.23 × 10^10^ gC year^−1^, assuming that the flux was 34.2 μmol C m^−2^ h^−1^ in the bay^47^. The annual DOC efflux from sediment pore water corresponded to 7% of the TOC sedimentation rate in the bay (3.1 × 10^10^ gC year^−1^).

**Figure 6:**
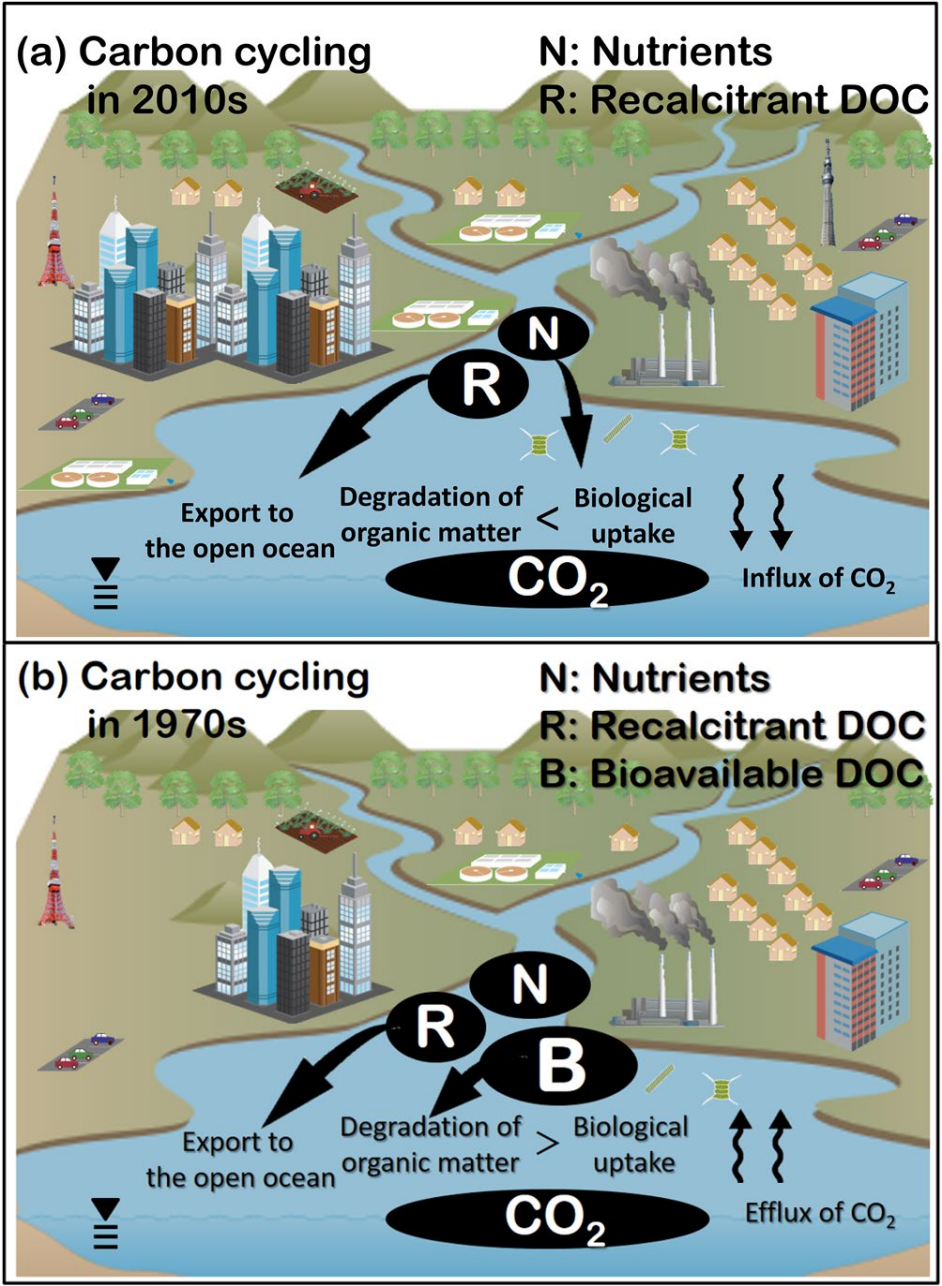
Carbon cycling model in Tokyo Bay in (**a**) 2011–2012 and (**b**) 1970s. Each carbon flux is shown in Table S4. The illustration was created by the image library, which was free material provided by Integration and Application Network, University of Maryland Center for Environmental Science (ian.umces.edu/imagelibrary/).

A large amount of terrestrial organic carbon flowed into Tokyo bay (4.9 × 10^1010^ gC year^−1^). The bioavailable DOC (BDOC), recalcitrant DOC (RDOC), and POC fluxes flowing into the bay were 1.0, 2.0, and 1.9 × 10^10^ gC year^−1^, respectively, which were estimated from the percentage of BDOC, RDOC, and POC to total organic carbon^26^ (20.4, 40.8, and 38.8%, respectively) multiplied with the total flux of organic carbon. Terrestrial BDOC was re-mineralized during the residence time of water in the bay^26^. Similarly, terrestrial POC was re-mineralized and deposited in the sediment during the water’s residence time^5^. Terrestrial RDOC was therefore suggested to be mainly exported to the open ocean based on the re-mineralization of terrestrial BDOC, the small BDOC fraction contributed by marine phytoplankton^26^, and POC re-mineralization and sedimentation (Fig. 6a, Table S4). In addition, the STPs in the watershed of Tokyo Bay removed organic carbon from freshwater sources. Tokyo Bay has consequently become a net sink for atmospheric CO_2_, because these processes have caused the nutrient supply to exceed the organic carbon supply^27^.

Figure 6b shows a conceptual carbon cycling model for Tokyo Bay during the 1970s, when the annual freshwater DIC, TOC, BDOC, RDOC, and POC loading was 9.1 × 10^10^, 13.1 × 10^10^, 2.3 × 10^10^^21^, 2.8 × 10^10^^21^, and 8.0 gC year^−1^^25^, respectively, and the average TOC sedimentation rate was 4.2 × 10^10^ gC year^−1^^22^. The GPP in the 1970s was approximately three times higher than that in 2011–2012^41^, which was likely related to the nutrient concentrations in Tokyo Bay being approximately twice as high in the 1970s than in 2011–2012^9,40^. However, the TOC sedimentation rate did not differ much between 2011–2012 and the 1970s, despite decreased organic carbon influxes into and primary production in the bay over the intervening period. Thus, we concluded that organic carbon was exported to the open ocean at higher levels during the 1970s than in recent years and/or that CO_2_ was outgassed to the atmosphere because of the degradation of labile DOC (Fig. 6b, Table S4).

Changes in carbon cycling between the 1970s and the present are largely due to sewage treatment in urbanized areas of the Tokyo Bay catchment. Improved sewage treatment with increasing urbanization has changed the quantity and quality of carbon flowing into the bay over time. The size of the urban area increased from 10.3% in 1972 to 23.5% in 2011, whereas the total area of cropland and forest decreased from 81.9% in 1972 to 61.3% in 2011^48^. In addition, STP effluent discharged into the bay increased by 48% between 1974 and 2012 (Japan Sewage Works Association). The POC and BDOC inputs were 2.3 and 4.2 times lower in 2011–2012 than in the 1970s, respectively, whereas RDOC was only 1.4 times lower. These large decreases in BDOC inputs into Tokyo Bay were similar to those in the Elbe estuary, Germany, where the installation of STPs around the watershed increased. Consequently, the partial pressure of CO_2_ in the Elbe estuary decreased from 7000 to 2500 μatm between 1986 and 2007^49^. Highly urbanized coastal waters in Guanabara Bay were also a significant net CO_2_ sink with high photosynthesis^4^.

Organic carbon fluxes in coastal waters increase in areas with increasing population and domestic wastewater effluent^14^. However, a decreasing trend has been reported in watersheds with high STP coverage and increased sewage treatment efficiency^50,51^. Biological oxygen demand also decreased in urbanized coastal waters because of increased STP effluent^51^. Consequently, BDOC may decrease in highly urbanized coastal waters because of increased STP installation and improved sewage treatment efficiency. The carbon flow change that support of obtained results in this study has been reported in world urbanized coastal waters. As a result, changes in carbon cycling may have occurred in highly urbanized coastal waters where STP coverage and sewage treatment efficiency have increased.

Thus, we postulated that the urbanization of coastal areas, such as Tokyo Bay, might have changed the coastal waters from a CO_2_ source to sink. Carbon cycling flows of Tokyo Bay in the 1970s were similar to the coastal carbon cycle recently reported in global coastal waters, because coastal waters are sources of BDOC to the open ocean^52,53^ and CO_2_ to the atmosphere^2,3^. Urbanized areas are expanding worldwide and is coincident with increasing sewage coverage^54^. Therefore, changes in carbon cycling similar to those observed in Tokyo Bay with increased coastal urbanization and improved sewerage can be expected globally. Carbon cycling could thus change dramatically, especially in coastal waters surrounded by megacities with ineffective wastewater systems. Continuous observation of the carbon budget around urbanized coasts will improve understanding of changes in the carbon cycle and ensure greater accuracy of carbon cycling modeling for future projections.

Carbon cycling changes in Tokyo Bay have occurred with the removal of organic matter and nutrients by the STPs in the basin. The bottom water was often anoxic (Fig. S1) because of organic matter decomposition between June and September. Large amounts of phosphate were released from the sediment in the bay during this period^20,47^, since high amounts of legacy phosphorus derived from past human activity accumulated^11^. However, the volume of anoxic water has recently been decreasing, although the volume has not changed significantly between the 1980s and 2000s^55^. Therefore, the bottom-water phosphate concentration decreased because decreased phosphate efflux from sediment^11^. This, in turn, decreased the surface phosphate concentration and limited primary production, because phosphate supply to the surface water decreased when surface water mixed with bottom water in autumn. Sustained wastewater treatment over the past 20 year may have contributed to decreased phosphate pools in Tokyo Bay. Similar to changes in the phosphorus cycle, those in the carbon cycle may also have occurred over recent decades.

## Conclusion

We estimated the carbon budget in highly urbanized coastal waters, namely Tokyo Bay, during 2011–2012. In addition, the carbon budget in the 1970s was estimated from the literature and compared with current data. Although carbon input from rivers during 2011–2012 was significantly lower than that in the 1970s, the TOC sedimentation rate did not change significantly. Organic carbon was probably exported to the open ocean at higher levels during the 1970s than in recent years, and/or CO_2_ has been released to the atmosphere because of the degradation of labile DOC. Changes in carbon cycling were largely a consequence of sewage treatment to remove labile organic carbon in urbanized areas of the Tokyo Bay catchment area.

## Supplementary information

Supplementary Information.

Supplementary Figures.
