# Supplementary material for: Coastal urbanization alters carbon cycling in Tokyo Bay
Source: Sci Rep. 2020 Nov 23;10:20413. doi: 10.1038/s41598-020-77385-4 (PMC7683726; doi:10.1038/s41598-020-77385-4)
Supplement: Supplementary file 1 — Supplementary Information. [file 41598_2020_77385_MOESM1_ESM.docx]

Coastal urbanization alters carbon cycling in Tokyo Bay

Atsushi Kubo1* and Jota Kanda2

1Department of Geosciences, Shizuoka University, 856 Ohya, Suruga-ku, Shizuoka city, Shizuoka 422-8529, Japan.

2Department of Ocean Sciences, Tokyo University of Marine Science and Technology, 4-5-7 Konan, Minato-ku, Tokyo 108-8477, Japan.

*Corresponding Author

Atsushi Kubo (kubo.atsushi@shizuoka.ac.jp; Telephone: +81-54-238-4794)

 Method of Box Model Analysis

A simple advective-diffusive box-model by Matsumura et al. (2002) was adopted to estimate the carbon budget in Tokyo Bay. In this model, Tokyo Bay was divided into two-layered boxes by three cross sections. The two layers were separated by an interface situated 7.5 m deep according to Matsumura et al. (2002). Three cross sections were divided into inner bay, central bay, and bay mouth region. In this model, mass balance for water, salinity, DIC, and TOC were calculated for box 1-4. The data from surface to 50 m for box 5 and 6 (station TB7 and TB8) were used to boundary data for each parameter.

The balance equations of freshwater and salt for each box were expressed as:

1. Box 1

|  | $A_{13}U_{13}=A_{12}W_{12}+Q_{W1}$ | (2) |
| --- | --- | --- |
|  |  |  |
|  | $V_{1}\frac{dS_{1}}{dt}+A_{13}U_{13}\frac{S_{1}+S_{3}}{2}-A_{13}K_{H13}\frac{S_{3}-S_{1}}{L_{13}}$  $=A_{12}W_{12}\frac{S_{1}+S_{2}}{2}+A_{12}K_{V12}\frac{S_{2}-S_{1}}{L_{12}}$ | (3) |

1. Box 2

|  | $A_{12}W_{12}=-A_{24}U_{24}$ | (4) |
| --- | --- | --- |
|  |  |  |
|  | $V_{2}\frac{dS_{2}}{dt}+A_{12}W_{12}\frac{S_{1}+S_{2}}{2}+A_{12}K_{V12}\frac{S_{3}-S_{1}}{L_{12}}$  $=-A_{24}U_{24}\frac{S_{2}+S_{4}}{2}+A_{24}K_{H24}\frac{S_{4}-S_{2}}{L_{24}}$ | (5) |

1. Box 3

|  | $A_{35}U_{35}=A_{13}U_{13}+A_{34}W_{34}+Q_{W3}$ | (6) |
| --- | --- | --- |
|  |  |  |
|  | $V_{3}\frac{dS_{3}}{dt}+A_{35}U_{35}\frac{S_{3}+S_{5}}{2}-A_{35}K_{H35}\frac{S_{5}-S_{3}}{L_{35}}$  $=A_{13}U_{13}\frac{S_{1}+S_{3}}{2}-A_{13}K_{H13}\frac{S_{3}-S_{1}}{L_{13}}+A_{34}W_{34}\frac{S_{3}+S_{4}}{2}+A_{34}K_{V34}\frac{S_{4}-S_{3}}{L_{34}}$ | (7) |

1. Box 4

|  | $-A_{24}U_{24}+A_{34}W_{34}=-A_{46}U_{46}$ | (8) |
| --- | --- | --- |
|  |  |  |
|  | $V_{4}\frac{dS_{4}}{dt}-A_{24}U_{24}\frac{S_{2}+S_{4}}{2}+A_{24}K_{H24}\frac{S_{4}-S_{2}}{L_{24}}+A_{34}W_{34}\frac{S_{3}+S_{4}}{2}$  $+A_{34}K_{V34}\frac{S_{4}-S_{3}}{L_{34}}$  $=-A_{46}U_{46}\frac{S_{4}+S_{6}}{2}+A_{46}K_{H46}\frac{S_{6}-S_{4}}{L_{46}}$ | (9) |

where A_ij_ is the area of the cross-section between box i and box j, U_ij_ and W_ij_ are the horizontal flow velocity and vertical flow velocity, respectively, Q_wi_ is the amount of freshwater discharge flowing into box i, V_i_ and S_i_ are the volume and average salinity of box i, dS_i_/dt is the rate of change of the mean salinity with respect to the time, K_Hij_ and K_Vij_ are the horizontal diffusivity coefficient and vertical diffusivity coefficient between box i and j, and L_ij_ is the distance between box i and j. K_H13_ was assumed to be equal to K_H24_, and K_H35_ was equal to K_H46_ according to Matsumura et al. (2002). Horizontal diffusivity was mainly dominated by tidal current; therefore, the square of K_H_ is proportional to tidal current (Tanimoto et al., 2001). The value of K_H_ was estimated from K_H35_=6.02×K_H13_ by Unoki et al. (1980). Vertical diffusivity, K_V34_, was estimated by K_V12_ and the density gradient ratio of box 3-4 and box 1-2 (Matsumura et al., 2002) because it was inversely proportional to density gradient (Tanimoto et al., 2001). Using the above eight equations, a linear equation with eight unknowns for U_ij_, W_ij_, K_Hij_, and K_Vij_ was solved.

The DIC budgets in each box were calculated according to the following equation.

1. Box 1

|  | $P_{1}=-R_{DX1}+V_{1}\frac{dC_{DX1}}{dt}+A_{13}U_{13}\frac{C_{DX1}+C_{DX3}}{2}-A_{13}K_{H13}\frac{C_{DX3}-C_{DX1}}{L_{13}}-A_{12}W_{12}\frac{C_{DX1}+C_{DX2}}{2}-A_{12}K_{V12}\frac{C_{DX2}-C_{DX1}}{L_{12}}+F_{DX1}$ | (10) |
| --- | --- | --- |

1. Box 2

|  | $P_{2}=V_{2}\frac{dC_{DX2}}{dt}+A_{24}U_{24}\frac{C_{DX2}+C_{DX4}}{2}-A_{24}K_{H24}\frac{C_{DX4}-C_{DX2}}{L_{24}}+A_{12}W_{12}\frac{C_{DX1}+C_{DX2}}{2}+A_{12}K_{V12}\frac{C_{DX2}-C_{DX1}}{L_{12}}$ | (11) |
| --- | --- | --- |

1. Box 3

|  | $P_{3}=-R_{DX3}+V_{3}\frac{dC_{DX3}}{dt}-A_{13}U_{13}\frac{C_{DX1}+C_{DX3}}{2}+A_{13}K_{H13}\frac{C_{DX3}-C_{DX1}}{L_{13}}+A_{35}U_{35}\frac{C_{DX3}+C_{DX5}}{2}-A_{35}K_{H35}\frac{C_{DX5}-C_{DX3}}{L_{35}}-A_{34}W_{34}\frac{C_{DX3}+C_{DX4}}{2}-A_{34}K_{V34}\frac{C_{DX4}-C_{DX3}}{L_{34}}+F_{DX3}$ | (12) |
| --- | --- | --- |

1. Box 4

|  | $P_{4}=V_{4}\frac{dC_{DX4}}{dt}-A_{24}U_{24}\frac{C_{DX2}+C_{DX4}}{2}+A_{24}K_{H24}\frac{C_{DX4}-C_{DX2}}{L_{24}}+A_{46}U_{46}\frac{C_{DX4}+C_{DX6}}{2}-A_{46}K_{H46}\frac{C_{DX6}-C_{DX4}}{L_{46}}+A_{34}W_{34}\frac{C_{DX3}+C_{DX4}}{2}+A_{34}K_{V34}\frac{C_{DX4}-C_{DX3}}{L_{34}}$ | (13) |
| --- | --- | --- |

where P_i_ is the DIC production in box i. Positive values are interpreted to be producing DIC in the water column via positive respiration, while negative values are interpreted as consuming DIC in the water column via positive organic matter production. R_DXi_ is the DIC supply from the land to box i, C_DXi_ is the average DIC concentrations in box i, dC_DXi_/dt is the rate of change of the mean DIC concentration with respect to time and F_DXi_ is the flux of carbon dioxide between air and box i. In this box model, we assumed that the bay was a steady state. The value of DIC production in the surface layer (Box 1 and 3) were treated as Net Community Production (NCP). The NCP was consistent with the sum of supply from the land, lateral transport, and air-sea exchange of CO_2_.

Next, the TOC budgets in each box were calculated according to the following equation:

1. Box 1

|  | $D_{1}=R_{TX1}-V_{1}\frac{dC_{TX1}}{dt}-A_{13}U_{13}\frac{C_{TX1}+C_{TX3}}{2}+A_{13}K_{H13}\frac{C_{TX3}-C_{TX1}}{L_{13}}+A_{12}W_{12}\frac{C_{TX1}+C_{TX2}}{2}+A_{12}K_{V12}\frac{C_{TX2}-C_{TX1}}{L_{12}}+A_{TX1}$ | (14) |
| --- | --- | --- |

1. Box 2

|  | $D_{2}=D_{1}-V_{2}\frac{dC_{TX2}}{dt}-A_{24}U_{24}\frac{C_{TX2}+C_{TX4}}{2}+A_{24}K_{H24}\frac{C_{TX4}-C_{TX2}}{L_{24}}-A_{12}W_{12}\frac{C_{TX1}+C_{TX2}}{2}-A_{12}K_{V12}\frac{C_{TX2}-C_{TX1}}{L_{12}}$ | (15) |
| --- | --- | --- |

1. Box 3

|  | $D_{3}=R_{TOC3}-V_{3}\frac{dC_{TX3}}{dt}+A_{13}U_{13}\frac{C_{TX1}+C_{TX3}}{2}-A_{13}K_{H13}\frac{C_{TX3}-C_{TX1}}{L_{13}}-A_{35}U_{35}\frac{C_{TX3}+C_{TX5}}{2}+A_{35}K_{H35}\frac{C_{TX5}-C_{TX3}}{L_{35}}+A_{34}W_{34}\frac{C_{TX3}+C_{TX4}}{2}+A_{34}K_{V34}\frac{C_{TX4}-C_{TX3}}{L_{34}}+A_{TX3}$ | (16) |
| --- | --- | --- |

1. Box 4

|  | $D_{4}=D_{3}-V_{4}\frac{dC_{TX4}}{dt}+A_{24}U_{24}\frac{C_{TX2}+C_{TX4}}{2}-A_{24}K_{H24}\frac{C_{TX4}-C_{TX2}}{L_{24}}-A_{46}U_{46}\frac{C_{TX4}+C_{TX6}}{2}+A_{46}K_{H46}\frac{C_{TX6}-C_{TX4}}{L_{46}}-A_{34}W_{34}\frac{C_{TX3}+C_{TX4}}{2}-A_{34}K_{V34}\frac{C_{TX4}-C_{TX3}}{L_{34}}$ | (17) |
| --- | --- | --- |

where D_i_ is the TOC sedimentation rate in box i. In this equation, positive values are interpreted as sedimentation to the lower box or sediment, while negative values are interpreted to be resuspension from the lower box or sediment. Q_TXi_ is the TOC supply from the land to box i, C_TXi_ is the mean TOC concentration in box i, dC_TXi_/dt is the rate of change of the mean TOC concentration with respect to time and A_TXi_ is the TOC supply from the rain.

Validity and uncertainties of box model

The spatially averaged concentrations of salinity and organic and inorganic carbon show considerably systematic variation corresponding to the seasonal variation in supplies from the land, atmospheric gas exchange, and the estuarine structure. Therefore, this analysis at least manages to capture the main seasonal features of estuarine hydrography and the behavior of the carbon cycling. Estimating the errors of U_ij_, W_ij_, K_Hij_ were about 10% using the box model (Matsukawa and Suzuki, 1985). In addition, maximum error of P_i_ and D_i_ become about 50% (Matsukawa and Suzuki, 1985). Moreover, in this study, analytical error of organic and inorganic carbon was within <5%. However, the calculated values of the carbon flow were not so different the observed values (Table 1 and Table S5). This is probably because the terms and properties involved in the calculations are so many that the averaging effect acting on the independent errors become large enough to decrease the real errors considerably. Thus, the box model can provide synoptic understanding of hydrography and carbon flow in coastal environment.

Reference

Central Environmental Council (2011) Basic direction of the 7th total water pollution load control scheme. Ministry of the Environmental Japan, 60 pp (in Japanese).

Kobayashi, J. (1960) Average composition and its features of Japanese rivers. Study of Agriculture (Nogaku Kenkyu), 48, 63–106 (in Japanese).

Matsukawa, Y., & Suzuki, T. (1985) Box model analysis of hydrography and behaviour of nitrogen and phosphorus in a eutrophic estuary. Journal of Ocenography Society of Japan, 41, 407-426.

Matsumoto, E. (1985) Budgets and residence times of nutrients in Tokyo Bay, In “Marine and Estuarine Geochemistry” (Editors, Sigkeo, A. C., & Hattori, A.), Lewis Publishers, Chelsea, pp. 127-136.

Matsumura, T., Ishimaru, T., & Yanagi, T. (2002) Nitrogen and phosphorus budgets in Tokyo Bay. Oceanography in Japan, 11, 613-630. (in Japanese with English abstract).

Ogura, N. (1975) Further studies on decomposition of dissolved organic matter in coastal seawater. Marine Biology, 31, 101-111.

Sanada et al. (1999) Estimation of sedimentation processes in Tokyo Bay using radionuclides and anthropogenic molecular markers. Geochemistry, 33, 123-138 (in Japanese with English abstract).

Shimizu, J., et al. (2005) Distributions of sedimentary POPs (Persistent Organic Pollutants) in Tokyo Bay results from the survey of POPs in Tokyo Bay in 2002. Reports of Hydrographic Research, 41, 35-49 (in Japanese with English abstract).

Tanimoto, T., Hoshika, A., Mishima, Y., & Yanagi, T. (2001). Budget of suspended materials and nutrients in Osaka Bay. Oceanography in Japan, 10, 397-412.

Unoki, S., Okazaki, M., & Nagasgima, H. (1980). Circulation flow and hydrographic conditions in Tokyo Bay, Technical Report of the Physical Oceanography Laboratory. The Institute of Physical and Chemical Research, 89pp.

Table S1

Temperature (^o^C), DOC (μmol L^-1^), POC (μmol L^-1^), and δ^13^C_POM_ (‰), DIC, and Chl *a* (μg L^-1^) at the lower Arakawa River station.

| Date | Temp.  (^o^C) | DOC  (μmol L^-1^) | POC  (μmol L^-1^) | δ^13^C_POM_  (‰) | DIC  (μmol L^-1^) | Chl *a*  (μg L^-1^) |
| --- | --- | --- | --- | --- | --- | --- |
| May-2011 | 23.6 | 283 | 165 | –32.6 | 1759 | 3.9 |
| Jun-2011 | 24.3 | 232 | 113 | –27.6 | 1544 | 9.9 |
| Jul-2011 | 24.2 | 398 | 81 | –25.5 | 1339 | 1.4 |
| Aug-2011 | 23.9 | 185 | 56 | –25.6 | 1244 | 1.1 |
| Sep-2011 | 27.1 | 155 | 79 | –29.2 | 1373 | 9.2 |
| Oct-2011 | 19.0 | 187 | 74 | –27.2 | 1258 | 4.7 |
| Nov-2011 | 17.4 | 246 | 283 | –25.1 | 1142 | 5.1 |
| Dec-2011 | 10.7 | 236 | 60 | –25.7 | 1536 | 2.0 |
| Jan-2012 | 7.0 | 261 | 142 | –27.9 | 1583 | 7.6 |
| Feb-2012 | 9.3 | 338 | 310 | –33.8 | 1711 | 42.3 |
| Mar-2012 | 14.2 | 320 | 258 | –33.1 | 1738 | 51.1 |
| Apr-2012 | 19.0 | 301 | 212 | –32.3 | 1764 | 56.8 |
| Average | 18.3 | 262 | 153 | –28.8 | 1499 | 16.3 |

Table S2

Temperature (^o^C), DOC (μmol L^-1^), POC (μmol L^-1^), and δ^13^C_POM_ (‰), DIC, and Chl *a* (μg L^-1^) at the lower Tamagawa River station.

| Date | Temp.  (^o^C) | DOC  (μmol L^-1^) | POC  (μmol L^-1^) | δ^13^C_POM_  (‰) | DIC  (μmol L^-1^) | Chl *a*  (μg L^-1^) |
| --- | --- | --- | --- | --- | --- | --- |
| May-2011 | 23.2 | 162 | 65 | –25.0 | 1110 | 5.4 |
| Jun-2011 | 26.9 | 167 | 47 | –25.4 | 1264 | 4.1 |
| Jul-2011 | 21.4 | 203 | 48 | –25.7 | 855 | 0.6 |
| Aug-2011 | 23.7 | 263 | 71 | –23.8 | 1245 | 4.3 |
| Sep-2011 | 25.5 | 193 | 70 | –23.1 | 1019 | 1.2 |
| Oct-2011 | 19.3 | 111 | 22 | –25.3 | 938 | 0.5 |
| Nov-2011 | 17.4 | 138 | 108 | –24.4 | 856 | 1.5 |
| Dec-2011 | 11.5 | 121 | 18 | –23.3 | 1018 | 0.4 |
| Jan-2012 | 9.0 | 143 | 42 | –25.7 | 1033 | 5.3 |
| Feb-2012 | 10.9 | 150 | 106 | –25.2 | 831 | 2.2 |
| Mar-2012 | 15.7 | 170 | 92 | –24.9 | 1074 | 5.3 |
| Apr-2012 | 20.5 | 190 | 78 | –24.6 | 1317 | 8.3 |
| Average | 18.8 | 168 | 64 | –24.7 | 1047 | 3.3 |

Table S3

Temperature (^o^C), DOC (μmol L^-1^), POC (μmol L^-1^), and δ^13^C_POM_ (‰), DIC, and Chl *a* (μg L^-1^) at the Shibaura sewage treatment plant effluent.

| Date | Temp.  (^o^C) | DOC  (μmol L^-1^) | POC  (μmol L^-1^) | δ^13^C_POM_  (‰) | DIC  (μmol L^-1^) | Chl *a*  (μg L^-1^) |
| --- | --- | --- | --- | --- | --- | --- |
| May-2011 | 27.6 | 409 | 71 | –24.0 | 1810 | 3.7 |
| Jun-2011 | 26.4 | 410 | 42 | –24.5 | 2218 | 0.8 |
| Jul-2011 | 27.9 | 351 | 38 | –24.5 | 2094 | 0.5 |
| Aug-2011 | 27.7 | 292 | 48 | –24.6 | 1545 | 2.2 |
| Sep-2011 | 28.7 | 366 | 83 | –29.7 | 1430 | 1.2 |
| Oct-2011 | 24.4 | 341 | 53 | –24.5 | 1291 | 0.8 |
| Nov-2011 | 20.5 | 189 | 76 | –24.5 | 1152 | 0.3 |
| Dec-2011 | 17.7 | 288 | 134 | –24.7 | 1475 | 0.6 |
| Jan-2012 | 14.9 | 387 | 191 | –24.8 | 1797 | 0.9 |
| Feb-2012 | 17.2 | 305 | 79 | –24.6 | 1364 | 0.1 |
| Mar-2012 | 19.7 | 373 | 71 | –24.0 | 1487 | 7.3 |
| Apr-2012 | 22.2 | 441 | 63 | –23.4 | 1610 | 14.4 |
| Average | 22.9 | 346 | 79 | –24.8 | 1606 | 2.7 |

Table S4

Carbon flow of Tokyo Bay in 1970s and 2011-2012 (×10^10^ gC year^-1^)

| Carbon flow | | In 2011-2012  (This study) | In 1970s |
| --- | --- | --- | --- |
| TOC sedimentation rate | | 3.1 | 4.2^1^ |
| TOC input from land | BDOC | 1.0 | 2.3^2^ |
|  | RDOC | 2.0 | 2.8^2^ |
|  | POC | 1.9 | 8.0^3^ |
| DIC input from land | | 11.2 | 9.2^4^ |
| Air-sea exchange of CO_2_ | | 5.0 | No Data |
| Carbon outflow to open ocean (DIC+TOC) | | 18.0 | No Data |

1; Matsumoto (1985), 2; Ogura (1975), 3; Central Environmental Council (2011), 4; Kobayashi (1960)

Table S5

TOC sedimentation rate in Tokyo Bay (×10^10^ gC year^-1^)

| Observation  year | Sedimentation rate | Method | Reference |
| --- | --- | --- | --- |
| 1980-1981 | 4.2 | ^210^Pb method | Matsumoto (1985) |
| 1997 | 5.0 | ^210^Pb method | Sanada et al. (1999) |
| 2002 | 4.9 | ^210^Pb method | Shimazu et al. (2005) |
| 2011 | 3.1 | Box Model | This Study |
