# Supplementary material for: Coastal urbanization alters carbon cycling in Tokyo Bay
Source: Sci Rep. 2020 Nov 23;10:20413. doi: 10.1038/s41598-020-77385-4 (PMC7683726; doi:10.1038/s41598-020-77385-4)
Supplement: Supplementary file 2 — Supplementary Figures. [file 41598_2020_77385_MOESM2_ESM.pdf]

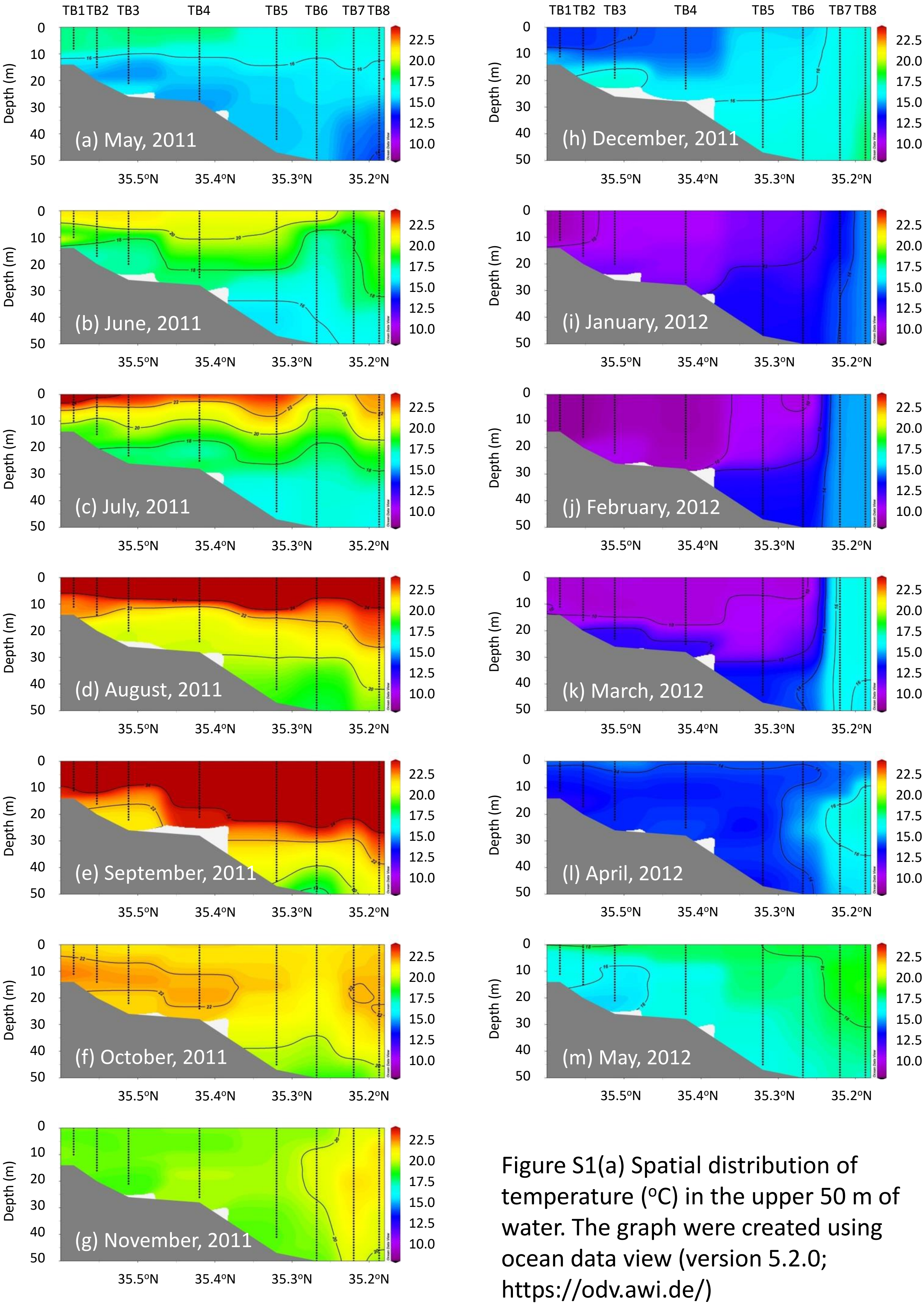

Figure S1(a) Spatial distribution of temperature (ºC) in the upper 50 m of water. The graph were created using ocean data view (version 5.2.0; https://odv.awi.de/)

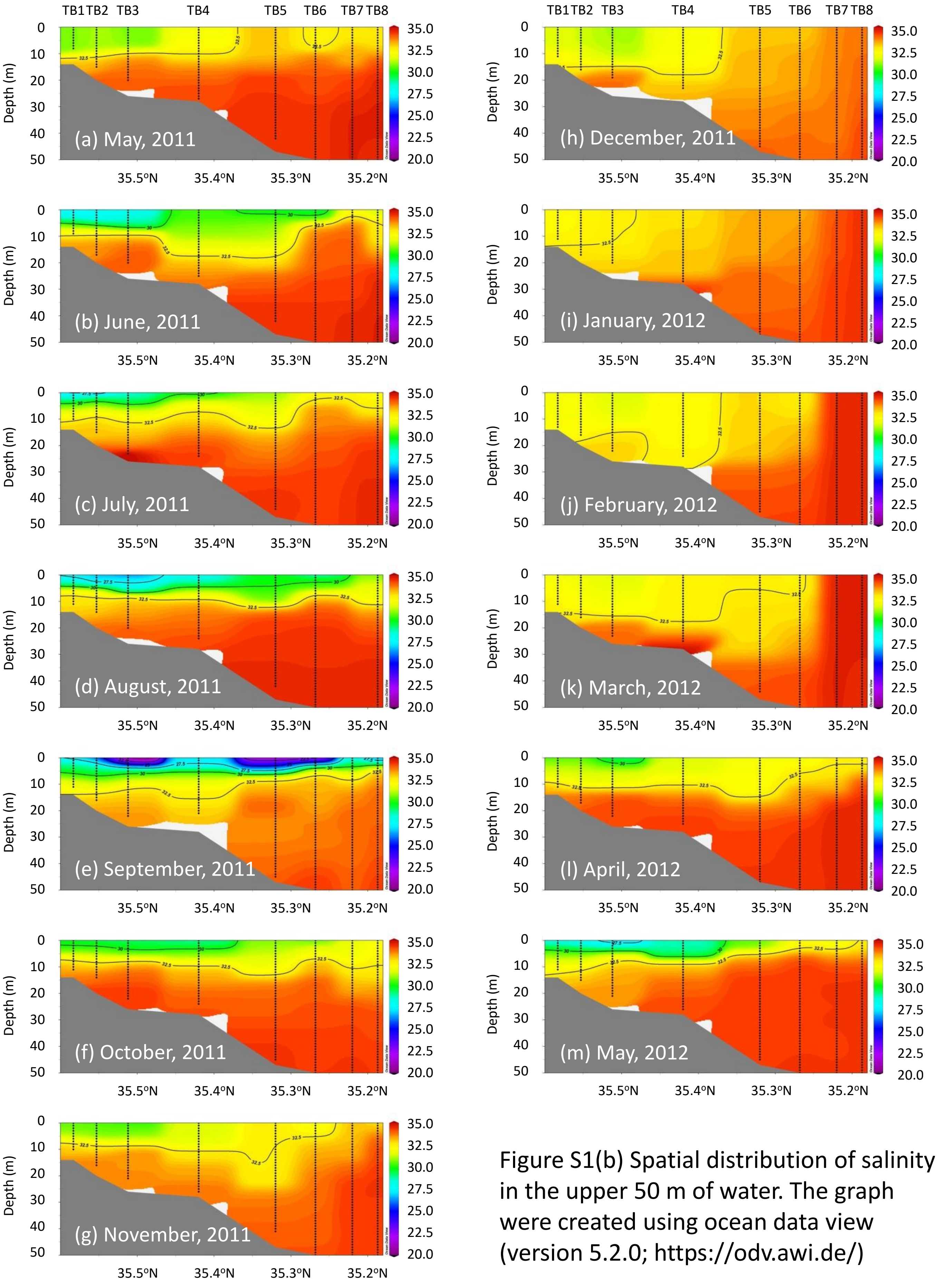

Figure S1(b) Spatial distribution of salinity in the upper 50 m of water. The graph were created using ocean data view (version 5.2.0; https://odv.awi.de/)

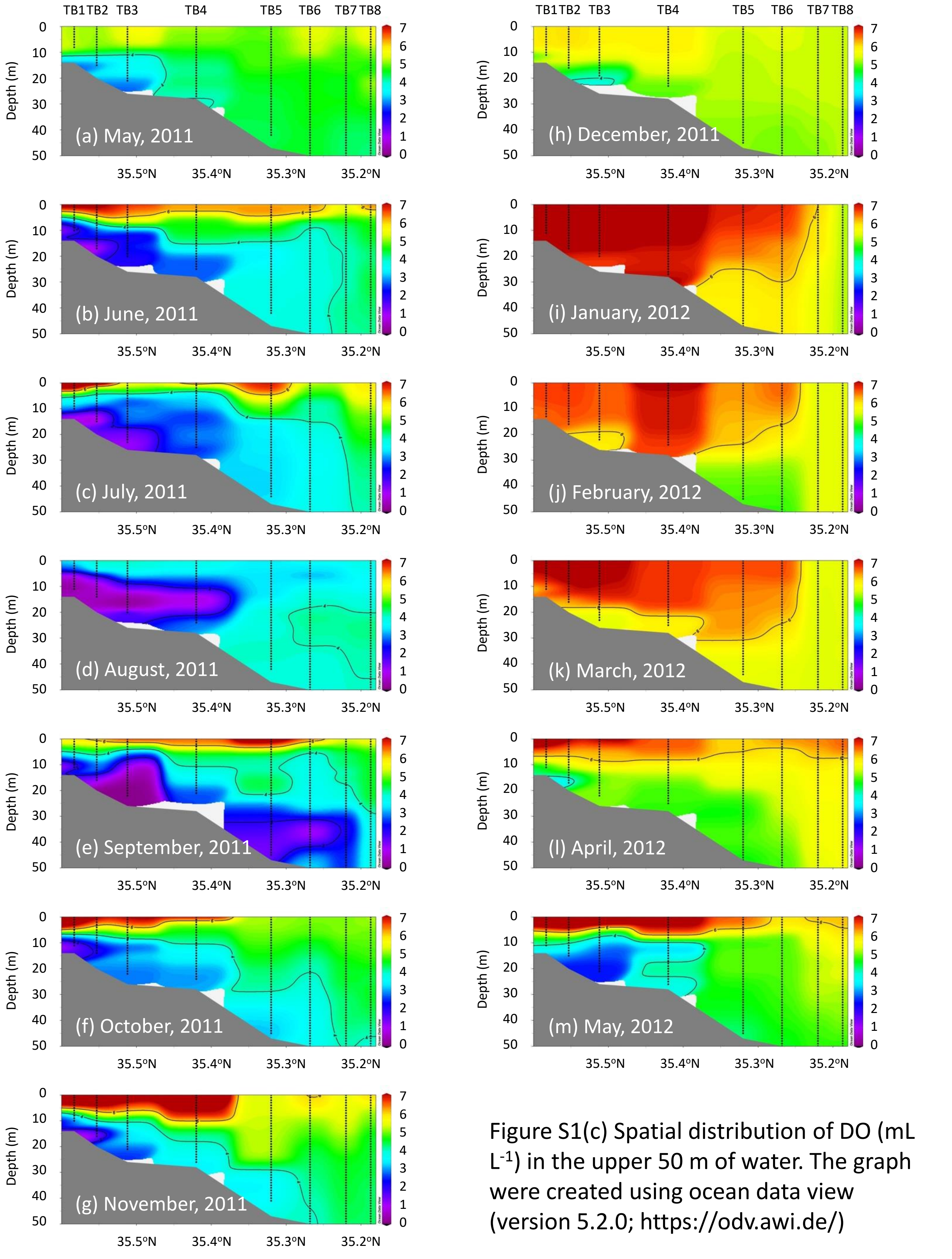

Figure S1(c) Spatial distribution of DO (mL L^{-1}) in the upper 50 m of water. The graph were created using ocean data view (version 5.2.0; https://odv.awi.de/)

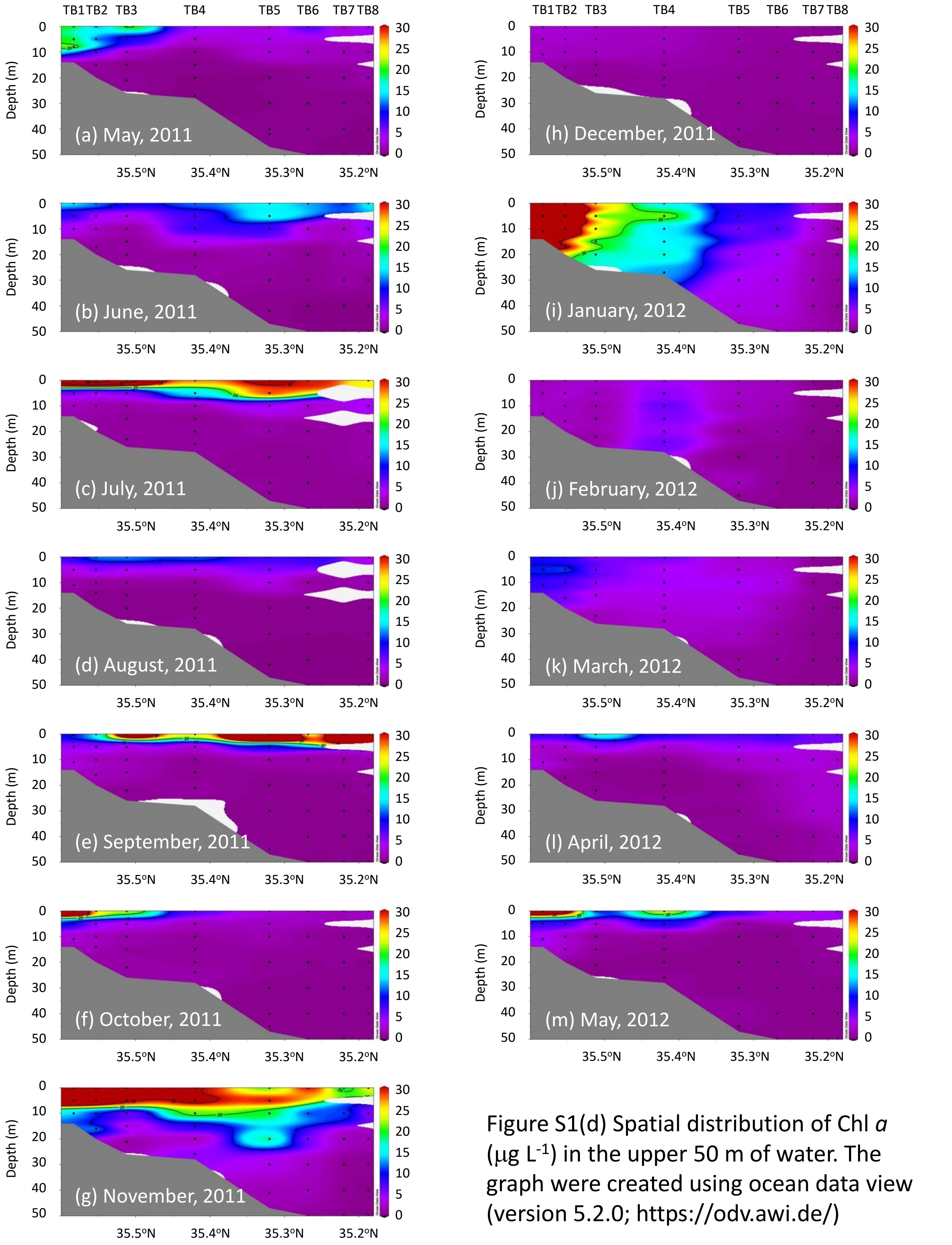

Figure S1(d) Spatial distribution of Chl *a* (μg L^{-1}) in the upper 50 m of water. The graph were created using ocean data view (version 5.2.0; https://odv.awi.de/)

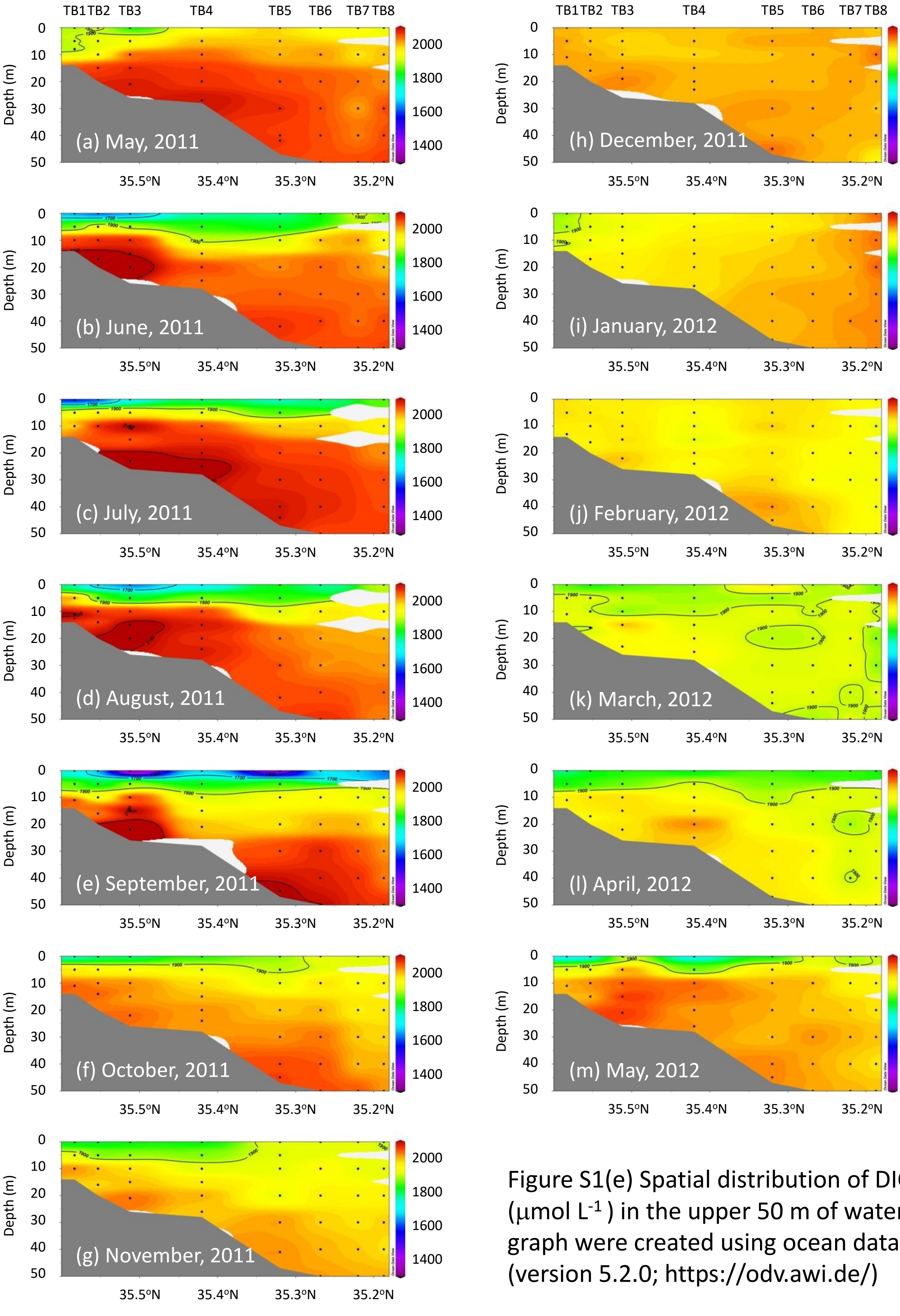

Figure S1(e) Spatial distribution of DIC (μmol L^{-1}) in the upper 50 m of water. The graph were created using ocean data view (version 5.2.0; https://odv.awi.de/)

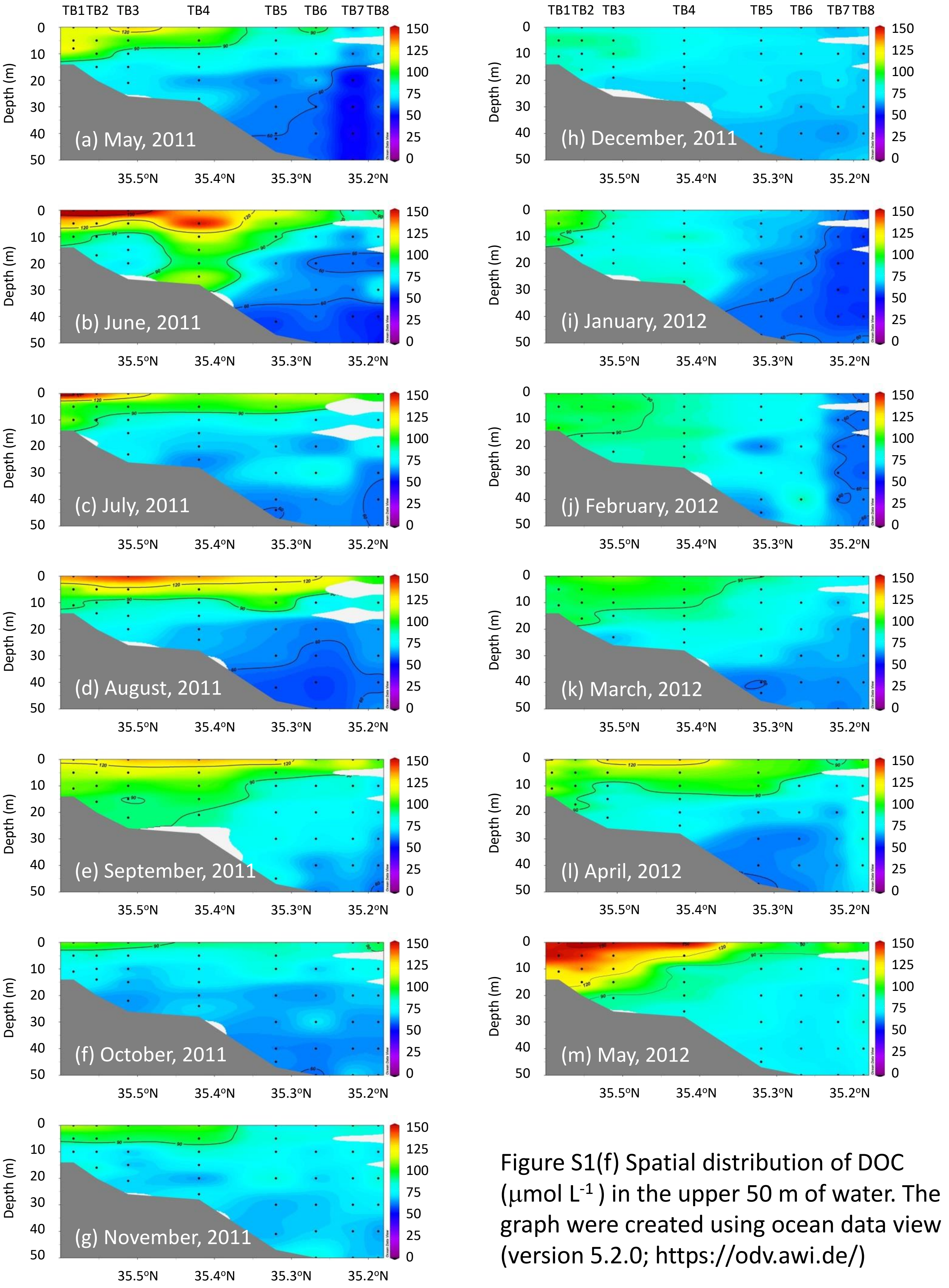

Figure S1(f) Spatial distribution of DOC (μmol L^{-1}) in the upper 50 m of water. The graph were created using ocean data view (version 5.2.0; https://odv.awi.de/)

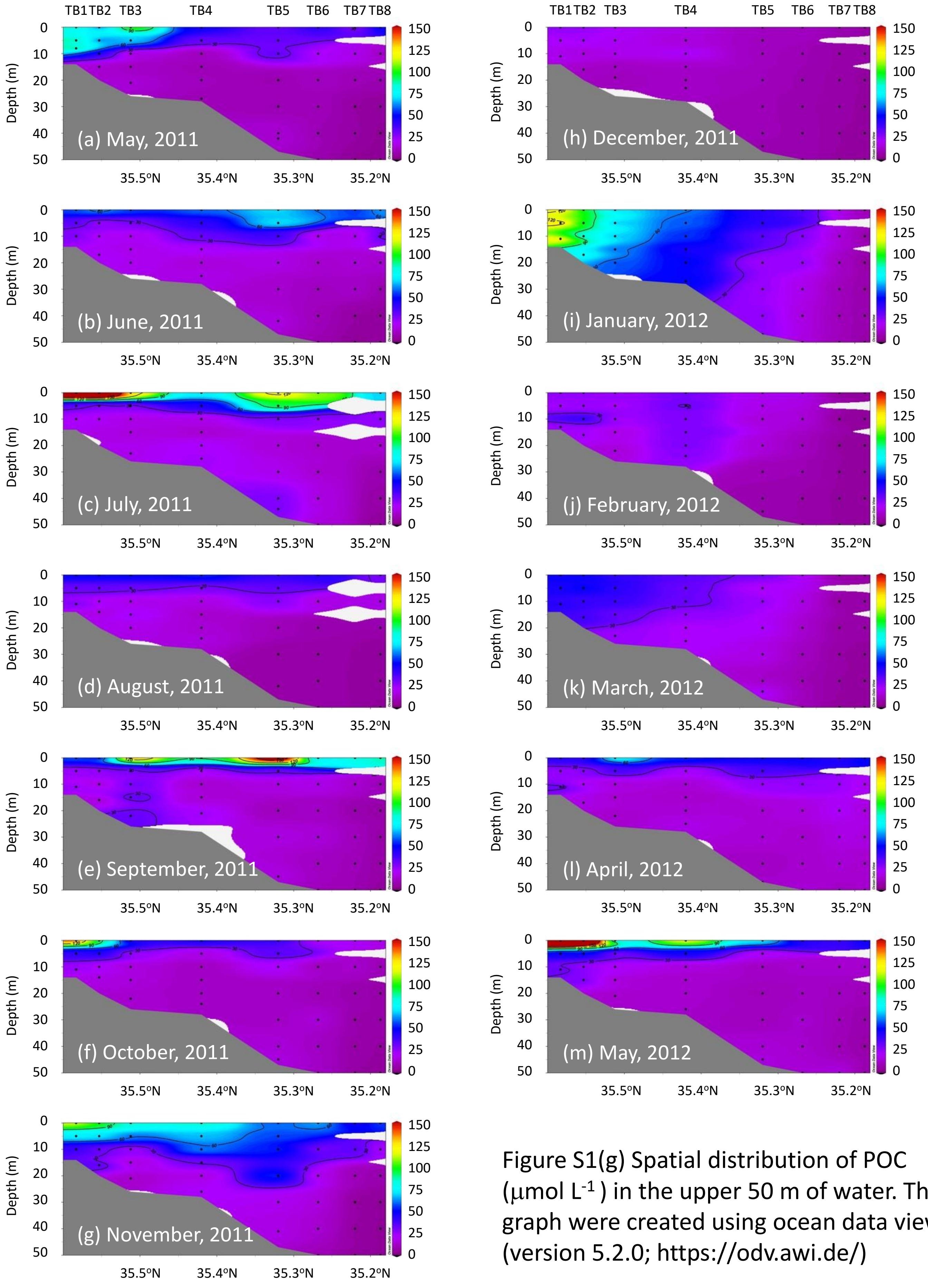

Figure S1(g) Spatial distribution of POC (μmol L[-1]) in the upper 50 m of water. The graph were created using ocean data view (version 5.2.0; https://odv.awi.de/)

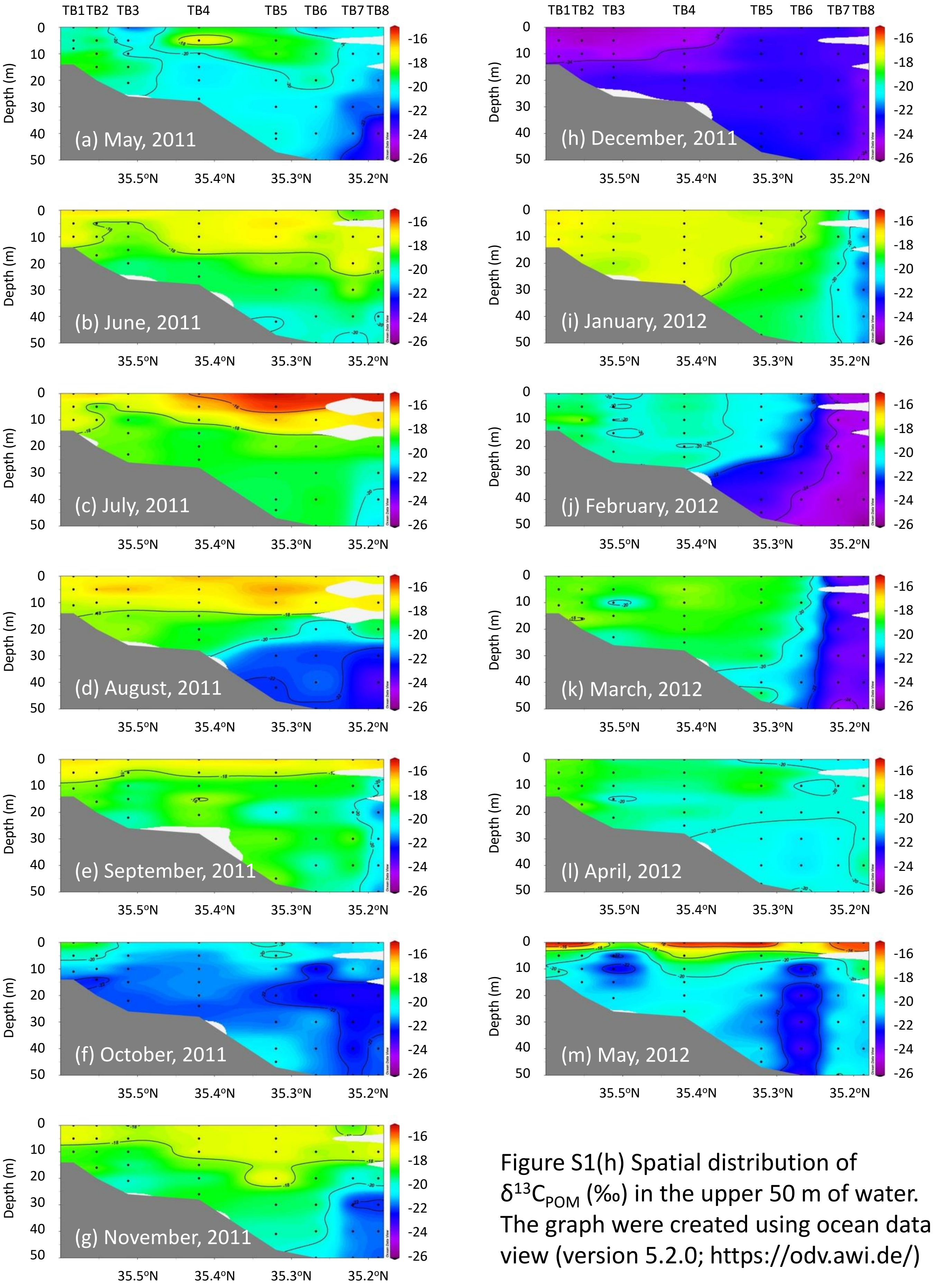

Figure S1(h) Spatial distribution of $\delta^{13}C_{POM}$ (‰) in the upper 50 m of water. The graph were created using ocean data view (version 5.2.0; https://odv.awi.de/)
